# Supplementary material for: Wbm0152, an outer membrane lipoprotein of the Wolbachia endosymbiont of Brugia malayi, inhibits yeast ESCRT complex activity
Source: bioRxiv. 2025 Jul 21:2025.07.21.665852. Preprint. [Version 1] doi: 10.1101/2025.07.21.665852 (PMC12330772; doi:10.1101/2025.07.21.665852)

bioRxiv preprint doi: https://doi.org/10.1101/2025.07.21.665852; this version posted July 25, 2025. The copyright holder for this preprint (which was not certified by peer review) is the author/funder, who has granted bioRxiv a license to display the preprint in perpetuity. It is made available under aCC-BY 4.0 International license.

Supplemental Information

Figure S1. wBm0152 does not bind other ESCRT subunits in vivo. SEY6210 strains harboring either the N-terminus (VN) or C-terminus (VC) of a Venus-YFP molecule on the C-terminus of the indicated ESCRT subunit were transformed with the corresponding copper-inducible pYES$_{CUP1}$-*w*Bm0152-VN or pYES$_{CUP1}$-*w*Bm0152-VC plasmid. Strains were grown in CSM media lacking uracil for 18h at 30°C with shaking, diluted 1:10 into fresh selective media lacking or supplemented with 0.5 mM $CuSO_4$, and outgrown for 6 hours before imaging. Bar = 5 µ; images are representative of three separate experiments.

bioRxiv preprint doi: https://doi.org/10.1101/2025.07.21.665852; this version posted July 25, 2025. The copyright holder for this preprint (which was not certified by peer review) is the author/funder, who has granted bioRxiv a license to display the preprint in perpetuity. It is made available under aCC-BY 4.0 International license.

Table S1. Primer and nucleotide sequences used in this study[a]

**Primers**		
YTDH F	5’-TACAGGGCGCGTGGGGATGATCCACTAGTATCATT ATCAATACTCGCCATTTCAAAG	pYES *TDH3* promoter conversion
YTDH R	5’-ATGATGATGATGATGATGAGAACCCCCCATGGTAA GCTTAATATTCCCTATAGTGAGTC	
CUP F	5’-TCTGTTAGAAACCTTGACTATGGG	pYES *CUP1* promoter conversion
CUP R	5’-GTTTCTCGGTCTAAGAGCTTATACG	
YCUP F	5’- TACAGGGCGCGTGGGGATGATCCACTAGTACCGAT CCCATTACCGACATTTG	
YCUP R	5’-ATGATGATGATGATGATGAGAACCCCCCATTTTAT GTGATGATTGATTGATTGATTGTAC	
0152VCN F	5’-GTAGAGTTTTCATTTTCTGGAATGAAAAAACGGAT CCCCGGGTTAATTAA	pYES-*w*Bm0152 split-Venus
0152VC R	5’-ATAACTAATTACATGATGCGGCCCTCTAGGCTACT TGTACAGCTCGTCCATGCC	
0152VN R	5’-ATAACTAATTACATGATGCGGCCCTCTAGGCTACT CGATGTTGTGGCGG	
VPS2 -500 F	5’-CGTAATACGACTCACTATAGGGCGAATTGGGTCAAA CAGCATTAAAAAGTGATATGC	pRS415-*VPS2* split-Venus
VPS2 VNC R	5’-GATTACGCCAAGCGCGCAATTAACCCTCACCCTAG CGGATCTGCCGGTAG	
VPS2VN F	5’-CGACTCACTATAGGGAATATTAAGCTTACCATGAG TTTGTTTGAGTGGGTATTTGG	pYES-*VPS2* split-Venus
VPS2VN R	5’-GAGACCGAGGAGAGGGTTAGGGATAGGCTTCTACTC GATGTTGTGGCGGATC	
BM6583 VN F	5’-CAAGCTAGGCTGGAAAATTTGAGACGTGAACGGAT CCCCGGGTTAATTA	BM6583 split-Venus
BM6583 VN R	5’- CCTATTATTCATTAAATATACTCAGAGCGCCTACT CGATGTTGTGGCGGA	
pr29	5’-CACATACGATTTAGGTGACAC	

bioRxiv preprint doi: https://doi.org/10.1101/2025.07.21.665852; this version posted July 25, 2025. The copyright holder for this preprint (which was not certified by peer review) is the author/funder, who has granted bioRxiv a license to display the preprint in perpetuity. It is made available under aCC-BY 4.0 International license.

pr32	5’-AATACGACTCACTATAGGGAG	*HPH* conversion
**gBlock®**		
yo*BM6583*-myc	5’-cgtaatacgactcactatagggcgaattgggCAGCTC<br>ATAAAGCATCTTAGTGAAAAGGGTGGTTTTGCGTTA<br>TTCTTTCCTCTGTTGAAGCTTTTCTATTTTGTTTAAGC<br>TGAACTAGTGGGAGTTTCCATTAAAAGGCTAAAGTC<br>TTGTTGAAACTTTTCCGGGTTAGATGATAACTGCGAA<br>AAGATAGATAAAATCAGCATTAAAGGACTTCGTCTC<br>CTCCTAATCAGAAATCATCCTTTCGAATTATGCGTATT<br>CAGTTGAAGCGTATTTGTGACCAGTGTACTTCAAGGC<br>TGATTGTTTCACCCTTGGACACAGAACGTTAAACA**ATG**<br>GACTTTCTGTTCGGGAGGAAGAAAACTCCTGCCGAG<br>ATGCTGAGACAAAACCAGAGGGCACTAAATAAGGCA<br>ATGAGAGAGCTTGATAGAGAGCGTTCAAGGCTAGAGA<br>TGCAAGAGAAAAAGATAATCGCAGATATAAAAAAGATG<br>GCTAAGATGAACCAGATGGACTCAGTAAAAGTAATGG<br>CTAAGGACCTTGTTAGGACAAGGCGTCATGTTAAAAAA<br>TTTATTATGATGAAAGCGAACATCCAGGCAGTCTCTTTG<br>AGAGTGCAGACTCTAAAATCTCAAGACGCCATGGCCC<br>AAGCGATGAAAGGTGTGACGAGGGCAATGCAGAATAT<br>GAATAGACAGCTTAATCTACCCCAGATCCAAAAAATCAT<br>GATGGAGTTCGAGCGTCAATCCGAGATTATGGATATGAA<br>GGAGGAAATGATGGGGGAAGCAGTTGATGATGCAATAG<br>CTGATGAAGGTGATGAGGAAGAGACCGAGACGATAGTC<br>GCCCAAGTTCTTGACGAACTAGGTATCCAGATGAACGAA<br>GAATTATCCGCAATCCCCGCAGCTCAGGGTTCTCTAAAA<br>CCTGCGGACCAGAACAGGCAACCTCAACCCGCTTTATC<br>AGACGCGGATGCTGATTTGCAAGCTAGGCTGGAAAATTT<br>GAGACGTGAAGAACAGAAGTTGATTTCCGAAGAAGACCT<br>C**TGA**GGATCCGCGCTCTGAGTATATTTAATGAATAATAGGT<br>CTATACTATAATACATCAACTACTGATATCAAAGTAGGCACT<br>TAGAATCAAAAGGCTTCCGAAAATTTGTTCAGTTTTTCACTT<br>TACTCAATCTCGTCACTAAAGTTTTCTTTTTTTTTCGAATGAAT<br>CCTCGAATAAATATGTTCTATATTATATATATACATCTTTTATATA<br>TCATTAAATGTACAGTAATCGGTCAAATTACAAATGCTTACGG<br>ATGATTTTTTCACTGATTAAGTACTCATAAATAATGGTAGCACA<br>TTCTTCAAgtgagggttaattgcgcgcttggcgtaatc	

[a]For gBlock® sequence, lowercase signifies regions of homology to pRS414, bolded sequence identifies *BM6583b* start codon, and underlined sequence is the appended BamHI restriction site immediately after stop codon (also bolded).

bioRxiv preprint doi: https://doi.org/10.1101/2025.07.21.665852; this version posted July 25, 2025. The copyright holder for this preprint (which was not certified by peer review) is the author/funder, who has granted bioRxiv a license to display the preprint in perpetuity. It is made available under aCC-BY 4.0 International license.

Figure S1.

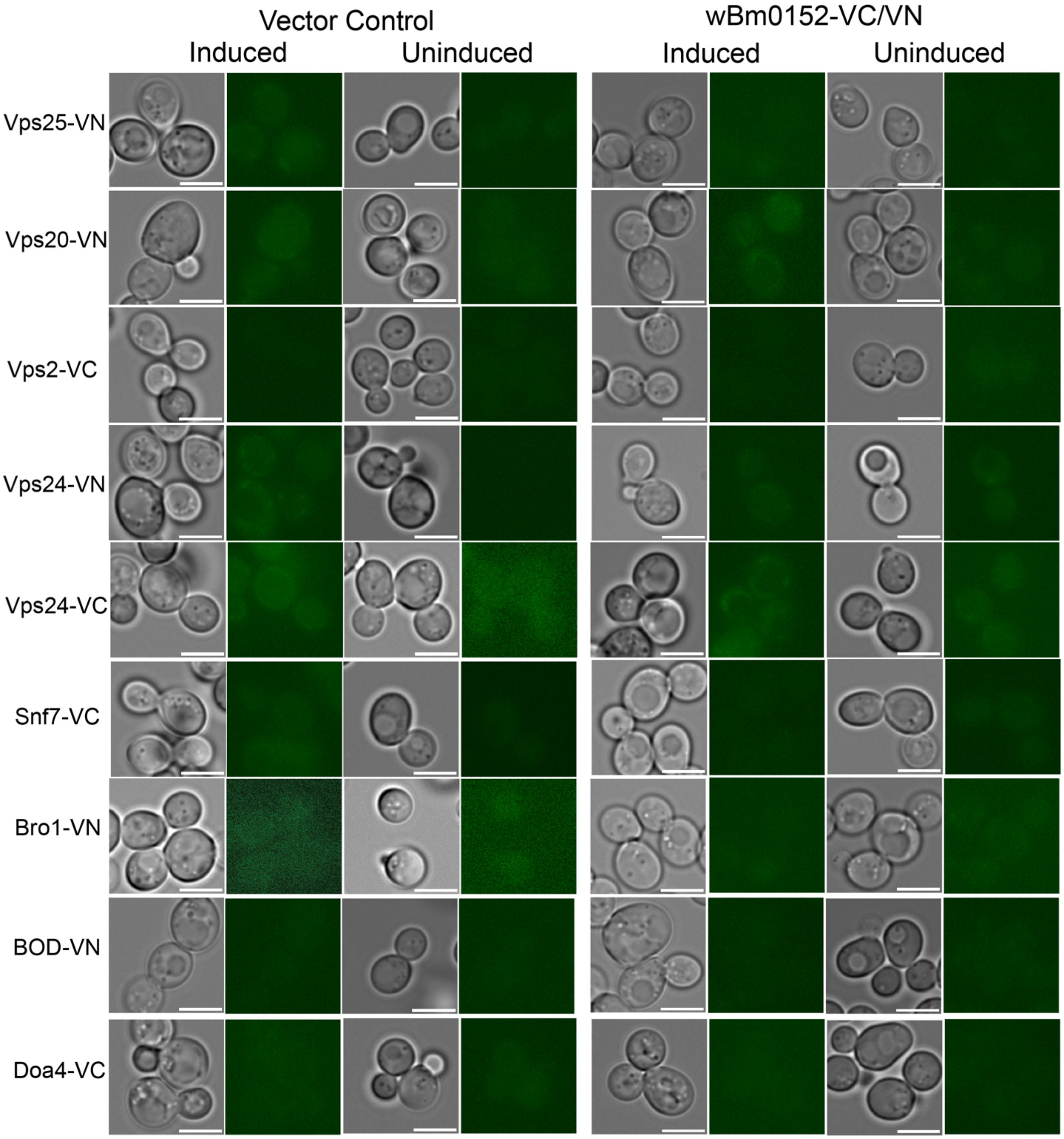

Supplement: Supplement 1 [file NIHPP2025.07.21.665852v1-supplement-1.pdf]
